# The Basic Psychological Need Satisfaction and Need Frustration at Work Scale: A Validation Study

**DOI:** 10.3389/fpsyg.2021.697306

**Published:** 2021-07-14

**Authors:** Anja Hagen Olafsen, Hallgeir Halvari, Claus Wiemann Frølund

**Affiliations:** School of Business, University of South-Eastern Norway, Hønefoss, Norway

**Keywords:** basic psychological needs, self-determination theory, need satisfaction, need frustration, autonomy, competence, relatedness, measurement

## Abstract

The aim of the present study was to adapt and validate the Basic Psychological Need Satisfaction and Frustration Scale within self-determination theory (SDT) within the work domain. Confirmatory factor analyses of three Norwegian samples and one English sample as well as multi-group analyses to examine measurement invariance were performed. The results showed that the adapted work-related scale with its six-factor structure fitted the data well in all four samples, and partial measurement invariance was obtained across samples and languages. Furthermore, internal consistencies for the subscales were acceptable and the subscales predicted work-related correlates as expected, demonstrating the criterion validity of the scale. The current study contributes to a unifying measurement for future research on one of the central underpinnings of SDT within the work domain.

## Introduction

One of the most prominent motivational theories of our time is self-determination theory (SDT; Deci and Ryan, 2000; Ryan and Deci, 2017), which posits that humans have inherent basic psychological needs that are the nutriments to human motivation, growth, flourishing, and well-being. In particular, basic psychological needs theory is one of six mini-theories within SDT that theorizes that when humans' basic psychological needs are satisfied humans thrive, while when these basic psychological needs are frustrated, maladjustment and even psychopathology can be the result (Vansteenkiste and Ryan, 2013; Vansteenkiste et al., 2020). Basic psychological needs are, hence, an essential part of the growing body of SDT research across various life domains, including that of work. Indeed, promoting need satisfaction (rather than need frustration) has been shown to be of essence for fostering autonomous work motivation, positive work attitudes and behaviors, as well as employee well-being (Deci et al., 2017; Olafsen and Deci, 2020).

A unified understanding and approach to the basic psychological needs is essential to accumulate research on this important mechanism within motivational research based on SDT. A measurement scale for basic psychological need satisfaction and frustration developed by Chen et al. (2015) has been an important piece in these efforts, and the Basic Psychological Need Satisfaction and Frustration Scale (BPNSFS) has become the go-to scale for assessing the mechanism of basic psychological needs within SDT. Currently, the scale has been adapted to various contexts, cultures, and languages. However, to the authors' knowledge, there is still no formal validation of this measurement scale in the work domain. Given the increased use of SDT in organizational psychology (Gagné and Deci, 2014), this scale would be fruitful to employ in future work. To have a common operationalization of basic psychological need satisfaction and frustration at work would enable the comparison of results both within and between contexts in the work domain. The purpose of the current study is to adapt the BPNSFS into a work domain measure and validate it in Norwegian and English. This contributes to (1) validation of the scale to fit the work context and (2) validation of the scale in Norwegian and English.

### Self-determination Theory

SDT (Deci and Ryan, 2000; Ryan and Deci, 2017) is a macro theory of human motivation that has developed through empirical work for the last six decades. Today, SDT represents a powerful framework of mini-theories that taps into different questions of human motivation and is used in a wide range of different contexts such as sports, education, health, and work. A central underpinning of SDT is the basic psychological needs. Specifically, within SDT it is acknowledged that all humans have a set of basic psychological needs that have to be satisfied for people to grow, flourish, and be physically and psychologically well. In contrast, if these needs are not satisfied, it will have physiological and psychological costs. Within SDT, three such basic psychological needs are identified through empirical work. First, the need for autonomy (deCharms, 1968) refers to the feeling of choice and concurrence with one's actions. Second, the need for competence (White, 1959), refers to the feeling of being effective and capable. Third, the need for relatedness (Baumeister and Leary, 1995) refers to the feeling of a connection to, caring for, and being cared for by other individuals and groups.

Decades of research point to the benefits of need satisfaction for quality of motivation, growth, functioning, and physical and psychological wellness across life domains (Ryan and Deci, 2017; Vansteenkiste et al., 2020). Moreover, in recent years, the concept of need frustration has gained increased attention, where research has shown detrimental consequences of getting the basic psychological needs for autonomy, competence, and relatedness frustrated (Bartholomew et al., 2011b; Chen et al., 2015; Olafsen et al., 2017). Importantly, need frustration is emphasized as a separate mechanism that leads to these outcomes, rather than being at the opposite pole of a need satisfaction continuum (Bartholomew et al., 2011b; Cordeiro et al., 2016). In particular, need frustration can be experienced when the basic psychological needs are actively undermined as a result of social contextual influences (Vansteenkiste et al., 2020)—autonomy when one is forced to undertake a certain task in a certain way, competence when one is told one cannot, or relatedness when one is being excluded or rejected. In this way, need frustration is something other than lack of need satisfaction, which represents a more passive obstruction of the basic psychological needs (e.g., not having choices, lacking skills, not sharing similar interest with a particular group). Need satisfaction and frustration is theorized to stand in an asymmetrical relation, where the absence of need satisfaction does not necessarily imply the presence of need frustration but where the presence of need frustration denotes the absence of need satisfaction (Vansteenkiste and Ryan, 2013). Following this, as an active threat of the basic psychological needs, need frustration has been shown to be an important predictor of detrimental consequences over and above satisfaction of these needs (Bartholomew et al., 2011a; Chen et al., 2015; Martinent et al., 2015).

### Basic Psychological Needs in the Workplace

Research building on SDT within organizational psychology has gained increased attention in the last 10–15 years. This body of research has addressed the links between motivation and the dual concerns of performance and employee well-being in organizations (Deci et al., 2017, Olafsen and Deci, 2020). A core element in much of this research is hence the support and satisfaction of the basic psychological needs for autonomy, competence, and relatedness for promoting quality work motivation, positive work functioning, and well-being. Accordingly, these needs have been used to study both antecedents and outcomes of motivational processes at work.

Based on this body of literature, a recent meta-analysis of 99 studies with 119 distinct samples examined the accumulated antecedents and consequences of basic psychological need satisfaction at work (Van den Broeck et al., 2016). The study showed that various social-contextual factors have been examined in relation to the basic psychological needs, such as job characteristics (various job resources and job demands) and organizational contexts (manager support, organizational support, justice, etc.). Furthermore, the meta-analysis pointed to various outcomes of well-being and job attitudes that have been the focus of previous studies. This goes to show the importance of the basic psychological needs for autonomy, competence, and relatedness as a central concept in understanding the motivational process going on in the workplace. In particular, basic psychological needs represent the psychological mechanisms that explain how and why social-contextual factors in the workplace are related to quality of motivation and various important work outcomes.

As for need frustration, the same meta-analysis concluded that this concept needs further research because it has not received much attention in the literature so far. However, some studies with a focus on basic psychological need frustration do exist. For instance, in a longitudinal study by Olafsen et al. (2017), the experience of need frustration at work was associated with higher levels of work-related stress, which predicted higher levels of somatic symptoms, emotional exhaustion, and absence due to sickness. Furthermore, Trépanier et al. (2015), showed how need frustration, as predicted by job demands (positive) and job resources (negative), related positively to controlled work motivation, which, in turn, related positively to psychological distress and psychosomatic complaints, and negatively to work engagement and work performance. As the last example, although other studies do exist, Van den Broeck et al. (2014) found a mediating role of frustration of the three basic psychological needs between job insecurity and counterproductive work behavior. While these findings show important results for the role of basic psychological need frustration at work, more research is still needed in this area. This requires adequate tools for assessing need frustration in combination with need satisfaction that can be used across studies in the work domain.

### Measurement of Basic Psychological Needs in the Work Context

Several measures have been developed and used to assess the basic psychological needs at work. For basic psychological need satisfaction, published work has typically used the Basic Need Satisfaction at Work scale, a 21-item questionnaire (Deci et al., 2001; Baard et al., 2004). This scale has been criticized for reasons related to lack of content validity, formal validation processes, and problems with reliability and intercorrelations for the subscales. More recent work has employed the Work-Related Basic Need Satisfaction Scale developed by Van den Broeck et al. (2010). While the Van den Broeck et al. (2010) scale has been argued to provide a better operationalization of basic psychological need satisfaction in the workplace because it followed traditional validation processes, past research has noted problems with this scale as well (Olafsen et al., 2015; Olafsen and Halvari, 2017; Tafvelin and Stenling, 2018). In particular, with the introduction of need frustration, it can be argued that some of the reversed score items might tap into need frustration rather than need satisfaction. As already mentioned, it has been acknowledged that these two concepts are not merely at the opposite ends of a continuum, rather being distinct concepts related to somewhat different antecedents and outcomes, and thus accounting for different motivational processes (Bartholomew et al., 2011a).

As a more recent concept within SDT, basic psychological need frustration in the work domain has so far mainly been assessed with an adapted version of the first measurement scale for need frustration developed by Bartholomew et al. (2011a), where, presumably, the scale is adapted to the work context [see for instance Olafsen et al. (2017), Silva et al. (2017)]. Moreover, Gillet et al. (2012) have adapted the same scale to work and validated it in French, and this has been used in several studies [see for instance Landry et al. (2016), Trépanier et al. (2016), Trépanier et al. (2015)]. In addition, a few studies have applied the Work-Related Basic Need Satisfaction Scale (Van den Broeck et al., 2010) to assess basic psychological need frustration. In particular, in the studies by Vander Elst et al. (2012) and Van den Broeck et al. (2014) the reversed score items for need satisfaction were used as indicators of need frustration. These measurements of need frustration have some challenges. First, the scale developed by Bartholomew et al. (2011a) has been criticized for also assessing antecedents of need satisfaction. Second, this scale has not been stringently validated in the work domain [with the potential exception of Gillet et al. (2012), but this is not readily available to most researchers because it is in French]. Third, as past studies have proven need satisfaction and need frustration to be distinct concepts, assessing need frustration using the same instrument as for need satisfaction is questionable.

In sum, there is a need for a more consistent measure to assess both satisfaction and frustration of the basic psychological needs within SDT in the work context. Recently, Longo et al. (2016) developed a scale assessing both need satisfaction and need frustration, hence tackling some of the issue with previous scales. This scale was validated in both the educational domain and in the work domain, with the latter being validated in one sample of MTurkers. Furthermore, Chen et al. (2015) developed a general scale to assess need satisfaction and need frustration—the BPNSFS. The BPNSFS has quickly become the scale of preference for the international community of SDT, with a lot of efforts being put into adapting it and validating it for different domains and in different languages. Indeed, these efforts are promising in terms of having a valid and reliable measure that works across cultures and life domains, including that of work. However, while the scale has been used to capture this concept in the work domain (Schultz et al., 2015), it has not yet been rigorously validated in this domain. Based on the support and attention given to the BPNSFS by the scholarly community, we believe we would be well-served by adding to this stream of studies by formally validating it within the work domain.

### The Present Study

This study sought to validate the Norwegian and English version of the BPNSFS adapted to the work domain in four steps. (1) The reliability and factorial validity of the Basic Psychological Need Satisfaction and Frustration at Work Scale (BPNSFWS) were examined in three Norwegian samples and one English sample. A three-factor model differentiating between items for each of the three basic psychological needs (both satisfaction and frustration items) was compared to a six-factor model differentiating between need satisfaction factors and need frustration factors [according to Chen et al. (2015)] using confirmatory factor analysis (CFA). (2), Measurement invariance (MI) across the samples using multi-group CFA, testing for configural, metric, scalar, and residual invariance (Putnick and Bornstein, 2016) was evaluated in two steps. First, we examined MI across the three Norwegian working samples. Second, we examined the MI between the Norwegian and the English scale. (3) The intercorrelations and reliability of the Norwegian and English subscales were then examined. (4) The criterion-related validity of the Norwegian and English scales was examined by looking at associations between the subscales and important antecedents and outcomes of satisfaction and frustration of the basic psychological needs in the literature. In line with SDT and previous research, we examined the associations between need satisfaction and frustration on one side and managerial need support, autonomous work motivation, controlled work motivation, vigor, emotional exhaustion, affective commitment, and turnover intention on the other. In particular, we expected positive associations between need satisfaction and managerial need support, autonomous work motivation, vigor, and affective commitment, while negative associations between need satisfaction and controlled motivation, emotional exhaustion, and turnover intention. For need frustration, we expected the opposite pattern of associations with the mentioned variables.

## Methods

### Procedures and Participants

Data were collected through online questionnaires. We used four samples to cover various occupations, organizations, and the Norwegian and English language (total *N* = 1,432) in the validation of the BPNSFWS. Sample 1 consisted of 281 employees in the finance and sales sector. Sample 2 consisted of 299 dental hygienists. Sample 3 consisted of 459 employees in a municipality. For samples 1 to 3, participants received an invitation to the survey through their work e-mail. In the invitation they were informed about the purpose and content of the study, the estimated time-usage, that their participation was voluntary, and that their answers would be completely confidential. Sample 4 consisted of 513 US employees reached through Amazon Mechanical Turk. The respondents were informed that participation was voluntary and anonymous and about the approximate response time. Responses were rewarded with $1,5 USD. In an effort to ensure high data quality, three analyses were performed. First, two cases were removed as duplicates from the same respondent. Second, 40 cases were removed for being completed in <5 min, indicating insufficient time dedicated to the task. Finally, 78 cases were removed for having impossible inconsistencies in the replies, for example by answering two directly opposing statements with “Completely agree.” This left a sample of 393 respondents. Samples 1, 2, and 3 were used to validate the Norwegian Scale, while sample 4 were used to validate the English scale. Demographics for the four samples are presented in Table 1. Approval was obtained from the Norwegian Center for Research Data prior to data collection (project numbers 578437, 53264, and 52866).

**Table 1:** Overview of demographic characteristics of the study samples.

	**Sample 1**	**Sample 2**	**Sample 3**	**Sample 4**
*N*	281	299	459	393
Gender				
Men	43.1%	2.0%	21.0%	47.6%
Women	54.4%	96.0%	76.0%	52.4%
Other/unknown	2.5%	2.0%	3.0%	0%
Age	Under 34 years: 27.6% 35–49 years: 44.0% 50 years and over: 28.4%	Under 20 years: 0.0% 20–29 years: 20.5% 30–39 years: 23.9% 40–49 years: 23.9% 50–59 years: 18.2% 60 years and over: 13.5%	Under 20 years: 0.7% 20–29 years: 9.3% 30–39 years: 21.2% 40–49 years: 32.1% 50–59 years: 26.9% 60 years and over: 9.9%	Under 20 years: 0.3% 20–29 years: 17.0% 30–39 years: 39.7% 40–49 years: 24.4% 50–59 years: 12.7% 60 years and over: 5.9%

### Measures

#### Need Satisfaction

Need satisfaction was measured using an adapted version of the BPNSFS (Chen et al., 2015) where the respondents are asked to rate their level of agreement with the various scale items, in this case, as they related to their personal experiences at work. The questionnaire adaptation process was guided by the suggestions of the International Test Commission (Bartram et al., 2018). First, the original scale was translated into Norwegian. This translated version was then back-translated, and the original scale was compared with this back-translation to ensure that the items reflected their original content. After verifying that the meaning of the items was not changed in the translated Norwegian version, the items in both Norwegian and English were adapted to fit the work context. The English version of the scale appears in Appendix B. The instrument consisted of subscales for autonomy satisfaction (four items; e.g., I feel that the decisions I make at work reflect what I really want), competence satisfaction (four items; e.g., I feel confident that I can do things well at work), and relatedness satisfaction (four items; e.g., I feel that the people I care about at work also care about me). The items were reported on a scale ranging from 1 (completely disagree) to 7 (completely agree).

#### Need Frustration

Need frustration was measured using an adapted version of the BPNSFS (Chen et al., 2015). The process was the same as described for the need of satisfaction part of the scale. The instrument consisted of subscales autonomy frustration (four items; e.g., I feel pressured to do many of the things I do at work), competence frustration (four items; e.g., I seriously doubt whether I can do things well at work), and relatedness frustration (four items; e.g., At work I feel excluded from the group that I want to be a part of). The items were reported on a scale ranging from 1 (completely disagree) to 7 (completely agree).

#### Managerial Need Support

Managerial need support was assessed in all four samples using the six-item version of the Work Climate Questionnaire (Baard et al., 2004). The items (e.g., I feel understood by my manager; α = 0.94, 0.94, 0.94, 0.94, ω = 0.94, 0.94, 0.95, 0.94 for samples 1, 2, 3, and 4, respectively), were measured on a scale ranging from 1 (completely disagree) to 7 (completely agree).

#### Motivation

The Norwegian and English version of the Multidimensional Work Motivation Scale (MWMS; Gagné et al., 2015) presented participants with the following stem in samples 2, 3, and 4: “I put effort into my job…” Participants rated preselected responses that assessed external regulation (six items; e.g. Because others will reward me financially only if I put enough effort into my job), introjected regulation (four items; e.g., Because I have to prove to myself that I can), identified regulation (three items; e.g., Because putting effort into this job has personal significance to me), and intrinsic motivation (three items; e.g., Because what I do in my work is exciting). Responses were made on a 7-point scale from 1 (not at all for this reason) to 7 (exactly for this reason). The scores on external regulation and introjection were added to make a composite for controlled work motivation (α = 0.85, 0.84, 0.82, ω = 0.83, 0.82, 0.81 for samples 2, 3, and 4, respectively), while identified regulation and intrinsic motivation were added to make a composite for autonomous work motivation (α = 0.90, 0.89, 0.92, ω = 0.89, 0.87, 0.92 for samples 2, 3, and 4, respectively).

#### Vigor

In samples 2, 3, and 4, the vigor subscale of the short version of the Utrecht Work Engagement Scale (Schaufeli et al., 2006) assessed vigor related to work (three items; e.g., At my work, I feel bursting with energy; α = 0.94, 0.89, 0.89, ω = 0.95, 0.89, 0.89 for samples 2, 3, and 4, respectively). Responses were made on a 7-point scale from 1 (never) to 7 (daily).

#### Emotional Exhaustion

In samples 2, 3, and 4, the emotional exhaustion subscale of the Maslach Burnout Inventory (Maslach et al., 1996) assessed emotional exhaustion at work (five items; e.g., I feel burned out from my work; α = 0.90, 0.91, 0.96, ω = 0.90, 0.91, 0.95 for samples 2, 3, and 4, respectively). Responses were made on a 7-point scale from 1 (never) to 7 (every day).

#### Affective Commitment

In samples 2, and 4, affective occupational commitment was assessed using the Affective Commitment Scale developed by Allen and Meyer (1990). The responses to the eight items (e.g., This occupation has a great deal of personal meaning for me; α = 0.84, 0.83, ω = 0.83, 0.89 for samples 2 and 4, respectively), were reported on a scale ranging from 1 (completely disagree) to 7 (completely agree).

#### Turnover Intention

Turnover intentions over the past year were assessed using three items based on the scale by Luchak and Gellatly (2007). A sample item is “The past year, I have regularly had an intention to leave” (α = 0.88, 0.93, 0.91, 0.96, ω = 0.87, 0.93, 0.91, 0.96 for samples 1, 2, 3, and 4, respectively). In samples 2 and 4, three additional items of current thinking about turnover from O'Driscoll and Beehr (1994) were assessed. A sample item is “I am thinking of leaving this job” (α = 0.92, 0.97, ω = 0.93, 0.97 for samples 2 and 4, respectively). Responses were made on a 7-point scale from 1 (never) to 7 (always).

### Data Analyses

The factor structure of the translated and adapted BPNSFWS was examined with CFA in Mplus (Muthén and Muthén, 1998–2017). Multi-group CFA was performed in Mplus to test for MI across the three Norwegian samples as well as between the Norwegian and the English version of the scale. MI was tested in four steps: (1) configural, (2) metric, (3) scalar, (4) residual (Putnick and Bornstein, 2016). further evaluation of convergent and discriminant validity was made on the basis of calculation of the Average Variance Extracted (AVE; Fornell and Larcker, 1981) and heterotrait-monotrait ratio (HTMT; Henseler et al., 2015) of correlations (i.e., the average of the correlations of indicators across constructs measuring different phenomena relative to the average of the correlations of indicators within the same construct) among the six scale dimensions. Internal consistency was evaluated by score reliability using SPSS Statistics 25 and JASP. Finally, zero-order correlations were used to establish criterion validity (Kline, 2005) between the subscales in the Norwegian and English BPNSFWS with related variables.

While Appendix A shows that the items are mostly normally distributed, a test of multivariate normality conducted by calculating Mardia's coefficient using the DeCarlo (1997) macro showed evidence of multivariate non-normality. That is, Mardia's normalized (i.e., standardized) coefficients of kurtosis of 46.935 in sample 1, 93.892 in sample 2, 89.147 in sample 3, and 100.473 in sample 4 were well-above the recommended cut-off of |3.0| suggested by Bentler and Wu (2002). Thus, the analyses were run with the robust maximum likelihood estimator to account for the non-normal data, and the Satorra–Bentler (S–B) scaled χ^2^ and robust standard errors adjustment to the maximum likelihood estimator are reported in the results section.

The fit of the models was evaluated using the chi-squared test (χ^2^), the root-mean-square error of approximation (RMSEA), the comparative fit index (CFI), and the standardized root-mean-square residual (SRMR) as recommended by Hu and Bentler (1999). While values for CFI above 0.95 are recommended (Hu and Bentler, 1999), values above 0.90 were deemed acceptable (Hoyle, 1995). Values below 0.08 were deemed acceptable for both SRMR and RMSEA (Hu and Bentler, 1999). MI was claimed acceptable if changes in the CFI were <0.01 coupled with changes in RMSEA <0.015 and SRMR <0.030 (metric invariance) or <0.015 (scalar and strict invariance) (Little, 2013). If the constrained model was rejected, a less restrictive model of partial invariance was evaluated in which, in accordance with modification indices and analysis of parameter estimates, equality constraints on one or more items were relaxed. If the model of partial invariance was accepted using these criteria, it was considered as the new reference model. For the remaining test of validity and reliability, values of Cronbach's alpha (α) and MacDonald's omega (ω) above 0.7 are typically deemed satisfactory for internal consistency (Nunnally, 1978), while AVE values of at least 0.50 (Fornell and Larcker, 1981) and HTMT ratio of correlation under 0.85 (Clark and Watson, 1995; Klein, 2015) or 0.90 (Gold et al., 2001; Teo et al., 2008) are common guidelines for convergent and discriminant validity, respectively.

## Results

### Factor Structure

For all samples, Table 2 goes to show that the six-factor model fitted the data better than the alternative three-factor model with acceptable fit indices for Sample 1^1^: χ^2^ (*df* = 232) = 433.36, *p* < 0.001, CFI = 0.93, SRMR = 0.060, and RMSEA = 0.057, 90% CI (0.049, 0.065); Sample 2^2^: χ^2^ (*df* = 233) = 344.86, *p* < 0.001, CFI = 0.96, SRMR = 0.062, and RMSEA = 0.041, 90% CI (0.032, 0.050); Sample 3^3^: χ^2^ (*df* = 230) = 414.66, *p* < 0.001, CFI = 0.95, SRMR = 0.052, and RMSEA = 0.043, 90% CI (0.037, 0.050); and Sample 4^4^: χ^2^ (*df* = 232) = 480.76, *p* < 0.001, CFI = 0.95, SRMR = 0.067, and RMSEA = 0.052, 90% CI (0.046, 0.059), respectively. Furthermore, all items had significant loadings (ranging from 0.60 to 0.90, *p* < 0.001, with an average loading of 0.77 in Sample 1; 0.51 to 0.94, *p* < 0.001, with an average loading of 0.78 in Sample 2; 0.58 to 0.86, *p* < 0.001, with an average loading of 0.74 in Sample 3; and 0.72 to 0.92, *p* < 0.001, with an average loading of 0.84 in Sample 4) on their intended latent factor (see Appendix B).

**Table 2:** Analyses of factor structure within the BPNSFWS across samples.

**Model**	**SBχ**^**2**^	***df***	***p***	**SCF**	**RSMEA**	**90% CI**	**CFI**	**SRMR**	**SBχ**^**2**^ **diff**	***df* diff**
3-factor model Sample 1	944.44	243	<0.001	1.2523	0.104	0.097–0.111	0.76	0.120	–	–
6-factor model Sample 1	425.57	231	<0.001	1.2380	0.056	0.048–0.064	0.93	0.059	429.35^***^	12
3-factor model Sample 2	703.48	245	<0.001	1.5335	0.082	0.075–0.089	0.83	0.096	–	–
6-factor model Sample 2	344.86	233	<0.001	1.5459	0.041	0.032–0.050	0.96	0.062	422.10^***^	12
3-factor model Sample 3	968.28	245	<0.001	1.4412	0.083	0.078–0.189	0.80	0.086	–	–
6-factor model Sample 3	462.23	233	<0.001	1.4446	0.048	0.042–0.055	0.94	0.053	529.20^***^	12
3-factor model Sample 4	2,086.96	244	<0.001	1.5298	0.137	0.132–0.142	0.66	0.190	–	–
6-factor model Sample 4	480.76	232	<0.001	1.5626	0.052	0.046–0.059	0.95	0.067	2725.79^***^	12

*** *p < 0.001*.

### Measurement Invariance Among the Norwegian Samples

#### Configural Invariance Test

To examine the configural MI of the measurement scale, a simultaneous multi-group CFA of the six-factor model was tested in the three Norwegian samples. This model (Model A) imposed no equality constraints on parameter estimates across groups. The results provided in Table 3 indicated acceptable fit for the tested model: χ^2^ (*df* = 697) = 1,225.34, *p* < 0.001, CFI = 0.943, SRMR = 0.057, and RMSEA = 0.048, 90% CI (0.044, 0.053).

**Table 3:** Invariance analysis of the BPNSFWS across the Norwegian samples.

**Model**	**SBχ**^**2**^	***df***	***p***	**SCF**	**RMSEA**	**90% CI**	**CFI**	**SRMR**	**SBχ**^**2**^ **diff**	***df*** **diff**	**ΔRMSEA**	**ΔCFI**	**ΔSRMR**
Model A	1,225.34	697	<0.001	1.4100	0.048	0.044–0.053	0.943	0.057	–	–	–	–	–
Model B	1,314.35	733	<0.001	1.4189	0.049	0.045–0.054	0.937	0.065	86.22^***^	36^a^	0.001	0.006	0.008
Model C	1,428.56	769	<0.001	1.4004	0.051	0.047–0.056	0.928	0.066	132.48^***^	36^b^	0.002	0.009	0.001
Model D	1,624.41	817	<0.001	1.4574	0.055	0.051–0.059	0.912	0.067	154.75^***^	48^c^	0.004	0.016	0.001
Model D2	1,565.51	815	<0.001	1.4547	0.053	0.049–0.057	0.919	0.066	117.16^***^	46^c^	0.002	0.009	0.000

*Model A: one-factor configural invariance (CI). Model B: one-factor CI and metric invariance (MI). Model C: one-factor CI, MI, and scalar invariance (SI). Model D: one-factor CI, MI, SI, and invariant uniquenesses (IU). Model D2: one-factor CI, MI, SI, and partial IU*.

*** *p <0.001*.

a *The reference model is Model A*.

b *The reference model is Model B*.

c *The reference model is Model C*.

#### Metric Invariance Test

The same model was tested simultaneously in the three Norwegian samples but constraining the corresponding item slopes to be equal across groups (Model B). The results provided in Table 3 indicated acceptable fit for the tested model: χ^2^ (*df* = 733) = 1,314.35, *p* < 0.001, CFI = 0.937, SRMR = 0.065, and RMSEA = 0.049, 90% CI (0.045, 0.054). Even though the constraints did cause a significant reduction in fit compared with Model A: S–B $\chi_{\text{diff}}^{2}$ = 82.22 (Δ*df* = 36), *p* < 0.001, it was considered acceptable because ΔCFI was <0.01, ΔRMSEA was <0.015, and ΔSRMR was <0.030.

#### Scalar Invariance Test

The same model was tested simultaneously in the three Norwegian samples but constraining both the corresponding item slopes and all the intercepts of the observed items to be equal across groups (Model C). The results provided in Table 3 indicated acceptable fit for the tested model: χ^2^ (*df* = 769) = 1,428.56, *p* < 0.001, CFI = 0.928, SRMR = 0.066, and RMSEA = 0.051, 90% CI (0.047, 0.056). Even though the constraints did cause a significant reduction in fit compared with Model B: S–B $\chi_{\text{diff}}^{2}$ = 132.48 (Δ*df* = 36), *p* < 0.001, it was considered acceptable because ΔCFI was <0.01, and ΔRMSEA and ΔSRMR were <0.015.

#### Invariant Uniqueness Test

A model adding cross-group equality constraints on all like items' residual variance was analyzed (Model D). The results provided in Table 3 indicated acceptable fit for the tested model: χ^2^ (*df* = 817) = 1,624.41, *p* < 0.001, CFI = 0.912, SRMR = 0.067, and RMSEA = 0.055, 90% CI (0.051, 0.059). Compared with Model C, the constraints did cause a significant reduction in fit: S–B $\chi_{\text{diff}}^{2}$ = 154.75 (Δ*df* = 48), *p* < 0.001 and ΔCFI was >0.01, while ΔRMSEA and ΔSRMR were <0.015. The modification indices suggested freely estimating the residual of item 2 for competence frustration. The new partial invariant uniqueness model (Model D2) showed acceptable fit to the data: χ^2^ (*df* = 815) = 1,565.51, *p* < 0.001, CFI = 0.919, SRMR = 0.066, and RMSEA = 0.053, 90% CI (0.049, 0.057). Even though the constraints did cause a significant reduction in fit compared with Model C: S–B $\chi_{\text{diff}}^{2}$ = 117.16 (Δ*df* = 46), *p* < 0.001, it was considered acceptable because ΔCFI was <0.01, and ΔRMSEA and ΔSRMR were <0.015.

### Measurement Invariance Between the Norwegian and the English Scale

Before testing the four-steps of measurement invariance of the six-factor model between the Norwegian and the English samples, the three Norwegian samples were combined into a single sample. Results of a CFA indicated acceptable fit for the tested model: χ^2^ (*df* = 231) = 637.10, *p* < 0.001, CFI = 0.951, SRMR = 0.043, and RMSEA = 0.043, 90% CI (0.039, 0.046)^5^.

#### Configural Invariance Test

To examine the configural MI of the measurement scale, a simultaneous multi-group CFA model was tested in between the combined Norwegian sample (group 1) and the English sample (group 2). This model (Model A) imposed no equality constraints on parameter estimates across groups. The results provided in Table 4 indicated acceptable fit for the tested model: χ^2^ (*df* = 461) = 1,052.65, *p* < 0.001, CFI = 0.957, SRMR = 0.051, and RMSEA = 0.043, 90% CI (0.040, 0.047).

**Table 4:** Invariance analysis of the BPNSFWS between the Norwegian and English scale.

**Model**	**SBχ**^**2**^	***df***	***p***	**SCF**	**RMSEA**	**90% CI**	**CFI**	**SRMR**	**SBχ**^**2**^ **diff**	***df* diff**	**ΔRMSEA**	**ΔCFI**	**ΔSRMR**
Model A	1,052.65	461	<0.001	1.5380	0.043	0.040–0.047	0.957	0.051	–	–	–	–	–
Model B	1,145.45	479	<0.001	1.5418	0.045	0.042–0.049	0.951	0.059	89.73^***^	18^a^	0.002	0.006	0.008
Model C	1,314.02	492	<0.001	1.5117	0.049	0.047–0.056	0.940	0.060	547.27^***^	13^b^	0.004	0.011	0.001
Model C2	1,263.57	491	<0.001	1.5132	0.048	0.045–0.051	0.944	0.060	392.86^***^	12^b^	0.003	0.007	0.001
Model D	1,433.03	520	<0.001	1.5621	0.051	0.048–0.054	0.933	0.062	136.61^***^	29^c^	0.003	0.011	0.002
Model D2	1,398.05	519	<0.001	1.5623	0.050	0.047–0.053	0.936	0.062	112.30^***^	28^c^	0.002	0.008	0.002

*Model A: one-factor configural invariance (CI). Model B: one-factor CI and metric invariance (MI). Model C: one-factor CI, MI, and scalar invariance (SI). Model C2: one-factor CI, MI, and partial SI. Model D: one-factor CI, MI, partial SI, and invariant uniquenesses (IU). Model D2: one-factor CI, MI, SI, and partial IU*.

*** *p <0.001*.

a *The reference model is Model A*.

b *The reference model is Model B*.

c *The reference model is Model C*.

#### Metric Invariance Test

The same model was tested simultaneously in the two groups but constraining the corresponding item slopes to be equal across groups (Model B). The results provided in Table 4 indicated acceptable fit for the tested model: χ^2^ (*df* = 479) = 1,145.45, *p* < 0.001, CFI = 0.951, SRMR = 0.059, and RMSEA = 0.045, 90% CI (0.042, 0.049). Even though the constraints did cause a significant reduction in fit compared with Model A: S–B $\chi_{\text{diff}}^{2}$ = 89.73 (Δ*df* = 18), *p* < 0.001, it was considered acceptable because ΔCFI was <0.01, ΔRMSEA was <0.015, and ΔSRMR was <0.030.

#### Scalar Invariance Test

The same model was tested simultaneously in the two groups samples but constraining both the corresponding item slopes and all the intercepts of the observed items to be equal across groups (Model C). The results provided in Table 4 indicated acceptable fit for the tested model: χ^2^ (*df* = 492) = 1,314.02, *p* < 0.001, CFI = 0.940, SRMR = 0.060, and RMSEA = 0.049, 90% CI (0.047, 0.056). The constraints did cause a significant reduction in fit compared with Model B: S–B $\chi_{\text{diff}}^{2}$ = 547.27 (Δ*df* = 13), *p* < 0.001 and ΔCFI was >0.01, while ΔRMSEA and ΔSRMR were <0.015. The modification indices suggested freely estimating the intercept of item 3 for relatedness satisfaction. The new partial invariant uniqueness model (Model C2) showed acceptable fit to the data: χ^2^ (*df* = 419) = 1,263.57, *p* < 0.001, CFI = 0.944, SRMR = 0.060, and RMSEA = 0.048, 90% CI (0.045, 0.051). Even though the constraints did cause a significant reduction in fit compared with Model B: S–B $\chi_{\text{diff}}^{2}$ = 392.86 (Δ*df* = 12), *p* < 0.001, it was considered acceptable because ΔCFI was <0.01, and ΔRMSEA and ΔSRMR were <0.015.

#### Invariant Uniqueness Test

A model adding cross-group equality constraints on all like items' residual variance was analyzed (Model D). The results provided in Table 4 indicated acceptable fit for the tested model: χ^2^ (*df* = 520) = 1,433.03, *p* < 0.001, CFI = 0.933, SRMR = 0.062, and RMSEA = 0.051, 90% CI (0.048, 0.054). Compared with Model C1, the constraints did cause a significant reduction in fit: S–B $\chi_{\text{diff}}^{2}$ = 136.61 (Δ*df* = 29), *p* < 0.001, and ΔCFI was >0.01, while ΔRMSEA and ΔSRMR were <0.015. The modification indices suggested freely estimating the residual of item 4 for relatedness frustration. The new partial invariant uniqueness model (Model D2) showed acceptable fit to the data: χ^2^ (*df* = 519) = 1,398.05, *p* < 0.001, CFI = 0.936, SRMR = 0.062, and RMSEA = 0.050, 90% CI (0.047, 0.053). Even though the constraints did cause a significant reduction in fit compared with Model B: S–B $\chi_{\text{diff}}^{2}$ = 112.30 (Δ*df* = 28), *p* < 0.001, it was considered acceptable because ΔCFI was <0.01, and ΔRMSEA and ΔSRMR were <0.015.

### Intercorrelations and Reliability

As displayed in Table 5, across the four samples, the latent variables of autonomy and competence satisfaction correlated on average 0.43, autonomy and relatedness satisfaction correlated on average 0.56, and competence and relatedness satisfaction correlated 0.34. Similarly, autonomy and competence frustration correlated on average 0.45, autonomy and relatedness frustration correlated on average 0.58, and competence and relatedness frustration correlated 0.58. As for the correlations between the subscales of autonomy, competence, and relatedness, autonomy satisfaction and autonomy frustration correlated on average −0.58, competence satisfaction and competence frustration correlated on average −0.64, and relatedness satisfaction and relatedness frustration correlated on average −0.61.

**Table 5:** AVE, HRMT ratio of correlations, and intercorrelations among scale dimensions across samples.

	**1. Autonomy satisfaction**				**2. Competence satisfaction**				**3. Relatedness satisfaction**				**4. Autonomy frustration**				**5. Competence frustration**				**6. Relatedness frustration**			
	**S1**	**S2**	**S3**	**S4**	**S1**	**S2**	**S3**	**S4**	**S1**	**S2**	**S3**	**S4**	**S1**	**S2**	**S3**	**S4**	**S1**	**S2**	**S3**	**S4**	**S1**	**S2**	**S3**	**S4**
1. Autonomy satisfaction	**0.64**	**0.54**	**0.55**	**0.72**	0.45	0.49	0.43	0.41	0.50	0.57	0.54	0.74	−0.59	−0.64	−0.55	−0.62	−0.37	−0.44	−0.40	−0.10	−0.45	−0.55	−0.47	−0.24
2. Competence satisfaction	0.46^***^	0.46^***^	0.41^***^	0.38^***^	**0.67**	**0.61**	**0.55**	**0.64**	0.27	0.37	0.28	0.46	−0.15	−0.33	−0.14	−0.38	−0.58	−0.69	−0.68	−0.63	−0.09	−0.24	−0.14	−0.55
3. Relatedness satisfaction	0.44^***^	0.55^***^	0.52^***^	0.72^***^	0.26^***^	0.36^***^	0.29^***^	0.44^***^	**0.60**	**0.64**	**0.56**	**0.81**	−0.25	−0.47	−0.32	−0.52	−0.14	−0.46	−0.28	−0.20	−0.52	−0.77	−0.65	−0.45
4. Autonomy frustration	−0.59^***^	−0.62^***^	−0.51^***^	−0.61^***^	−0.14^*^	−0.21^***^	−0.13^*^	−0.41^***^	−0.22^**^	−0.44^***^	−0.35^***^	−0.53^***^	**0.60**	**0.58**	**0.49**	**0.66**	0.25	0.42	0.49	0.44	0.32	0.58	0.55	0.61
5. Competence frustration	−0.38^***^	−0.41^***^	−0.38^***^	−0.07	−0.60^***^	−0.64^***^	−0.69^***^	−0.64^***^	−0.15^*^;	−0.42^***^	−0.30^***^	−0.19^***^	0.50^***^	0.30^***^	0.53^***^	0.47^***^	**0.47**	**0.68**	**0.54**	**0.73**	0.28	0.49	0.48	0.84
6. Relatedness frustration	−0.45^***^	−0.56^***^	−0.41^***^	−0.20^***^	−0.09	−0.23^***^	−0.14^*^	−0.54^***^	−0.52^***^	−0.80^***^	−0.69^***^	−0.42^***^	0.57^***^	0.57^***^	0.57^***^	0.62^***^	0.53^***^	0.49^***^	0.46^***^	0.84^***^	**0.59**	**0.68**	**0.65**	**0.72**

*S, Sample. AVE reported on, HTMT ratio of correlations reported above, and intercorrelations among scale dimensions reported below the diagonal*.

*** *p < 0.001*,

** *p < 0.01*,

* *p < 0.05*.

*The bold values on the diagonal are AVE*.

To further evaluate convergent and discriminant validity, AVE and HTMT ratio was calculated. Results showed that AVE for each of the six need dimensions had an AVE of 0.5 or above (Fornell and Larcker, 1981) across the fours samples. Two exceptions were found; for autonomy frustration in sample 3 (AVE = 0.49) and for competence frustration in sample 1 (AVE = 0.47). However, these represent quite small deviation from the recommendation and only appear in one sample and was deemed acceptable. As for the HTMT ratio, none were above the strictest threshold of 0.85 (Clark and Watson, 1995; Klein, 2015) indicating discriminant validity for the six scale dimensions.

Cronbach's alpha (α) and McDonald's ω were calculated for each subscale and showed adequate reliability with an average α = 0.86; ω = 0.86 for autonomy satisfaction, α = 0.86; ω = 0.86 for competence satisfaction, α = 0.87; ω = 0.87 for relatedness satisfaction, α = 0.86; ω = 0.86 for autonomy frustration, α = 0.86; ω = 0.85 for competence frustration, and α = 0.89; ω = 0.89 for relatedness frustration across the four samples (see Appendix B).

### Criterion-Related Validity

The correlations between the six sub-dimensions of the BPNSFWS and the criterion-related variables are presented in Table 6. As expected, autonomy, competence, and relatedness need satisfaction were positively associated with managerial need support, autonomous work motivation, vigor, and affective commitment, while negatively associated with emotional exhaustion and turnover intention. However, need satisfaction of autonomy, competence, and relatedness were not significantly associated with controlled work motivation. Rather, need frustration was a better predictor of controlled work motivation despite some inconsistencies between the samples. There was one exception of this, as the English sample showed a positive correlation between autonomy need satisfaction and controlled work motivation, and between relatedness need satisfaction and controlled work motivation. Additional analysis revealed that this association was caused by a positive correlation to introjection, in particular the introjection approach items (Gagné et al., 2015).

**Table 6:** Zero-order correlations among need satisfaction, need frustration, and work-related correlates across samples.

	**Autonomy satisfaction**				**Competence satisfaction**				**Relatedness satisfaction**			
	**S1**	**S2**	**S3**	**S4**	**S1**	**S2**	**S3**	**S4**	**S1**	**S2**	**S3**	**S4**
Managerial need support	0.60^***^	0.61^***^	0.52^***^	0.71^***^	0.26^***^	0.21^***^	0.22^***^	0.44^***^	0.39^***^	0.36^***^	0.35^***^	0.69^***^
Autonomous motivation		0.44^***^	0.41^***^	0.70^***^		0.45^***^	0.24^***^	0.35^***^		0.37^***^	0.33^***^	0.63^***^
Controlled motivation		−0.09	0.06	0.29^***^		−0.04	−0.05	0.04		0.04	0.03	0.29^***^
Emotional exhaustion		−0.37^***^	−0.31^***^	−0.41^***^		−0.25^***^	−0.12^*^	−0.36^***^		−0.29^**^	−0.14^**^	−0.39^***^
Vigor		0.48^***^	0.47^***^	0.62^***^		0.32^***^	0.32^***^	0.39^***^		0.29^***^	0.32^***^	0.62^***^
Affective commitment		0.31^***^		0.66^***^		0.21^***^		0.34^***^		0.23^***^		0.66^***^
Turnover intentions	−0.46^***^	−0.42^***^	−0.38^***^	−0.50^***^	−0.14^*^	−0.28^***^	−0.09	−0.31^***^	−0.21^***^	−0.38^***^	−0.29^***^	−0.45^***^
	**Autonomy frustration**				**Competence frustration**				**Relatedness frustration**			
Managerial need support	−0.41^***^	−39^***^	−0.39^***^	−0.48^***^	−0.23^***^	−22^***^	−0.22^***^	−0.19^***^	−0.40^***^	−0.39^***^	−0.40^***^	−0.35^***^
Autonomous motivation		−0.36^***^	−0.23^***^	−0.44^***^		−0.41	−0.20^***^	−0.07		−0.23^***^	−0.24^***^	−0.20^***^
Controlled motivation		0.16^**^	0.09	0.05		0.17^**^	0.21^***^	0.28^***^		0.10	0.18^***^	0.17^**^
Emotional exhaustion		0.51^***^	0.40^***^	0.59^***^		0.26^***^	0.29^***^	0.48^***^		0.33^***^	0.28^***^	0.49^***^
Vigor		−0.43^***^	−0.33^***^	−0.48^***^		−0.30^***^	−0.32^***^	−0.15^**^		−0.22^***^	−0.31^***^	−0.26^***^
Affective commitment		−0.24^***^		−0.54^***^		−0.19^**^		−0.24^***^		−0.17^**^		−0.39^***^
Turnover intentions	0.39^***^	0.51^***^	0.39^***^	0.57^***^	0.31^***^	0.26^***^	0.19^***^	0.40^***^	0.36^***^	0.39^***^	0.31^***^	0.49^***^

*S, Sample*.

*** *p < 0.001*,

** *p < 0.01*,

* *p < 0.05*.

Furthermore, as expected, frustration of the basic psychological needs for autonomy, competence, and relatedness was negatively associated with managerial need support, autonomous work motivation, vigor, and affective commitment, while positively associated with emotional exhaustion and turnover intention.

## Discussion

The purpose of the present study was to validate the BPNSFWS, which is based on the theoretical framework of SDT (Deci and Ryan, 2000; Ryan and Deci, 2017). The results across three samples of a total of 1,432 employees provided acceptable support for the properties of the adapted scale. In particular, consistent with SDT, results showed support for a six-factor structure of the BPNSFWS where satisfaction and frustration of the basic psychological needs for autonomy, competence, and relatedness were distinct factors (Chen et al., 2015). This factorial structure held across three different Norwegian occupational samples and one English sample. Calculations of AVE and HTMT ratios of correlations further supported the validity of the measurement scale. Furthermore, the subscales for each need proved to have satisfactory internal consistency. With these results, the present study contributes to establishing an important tool for a central concept within SDT for future studies of organizational psychology based on this theoretical framework. An important feature of the BPNSFWS is that it was adapted and validated specifically for the work context. Moreover, it addresses need satisfaction and need frustration as separate dimensions. Because previous research has employed domain-general scales to measure the basic psychological needs and/or scales with notable limitations such as content validity and lack of rigorous validation, the adapted scale enables researchers to study need satisfaction and frustration at work with items that are relevant in the work context and meaningful to employees and, therefore, have a better chance of generating reliable and valid scores of employee need satisfaction and frustration at work.

The associations of need satisfaction and frustration with common antecedents and outcomes within the SDT literature in the work domain generally provided criterion-related validity of the adapted scale. In particular, satisfaction of the three basic psychological needs was found to be positively related to positive work correlates (i.e., managerial need support, autonomous work motivation, vigor, and affective commitment) and negatively related to negative work correlates (i.e., emotional exhaustion and turnover intention). Conversely, frustration of the basic psychological needs was found to be negatively related to positive work correlates (i.e., managerial need support, autonomous work motivation, vigor, and affective commitment) and positively related to negative work correlates (i.e., controlled work motivation, emotional exhaustion, and turnover intention). In the English sample, albeit less strongly than to autonomous work motivation, autonomy and relatedness need satisfaction had a positive correlation to controlled work motivation. As this was caused by a positive correction to introjection, it follows previous findings for associations to the regulations in the MWMS where introjection also previously has been found to relate to “positive” variables (Gagné et al., 2015).

Because most previous research has focused on the bright path of motivational processes at work (Deci et al., 2017), the validation of the BPNSFWS is of particular importance for future studies on the dark side of these processes by providing a valid instrument to assess this path. As seen from the correlations in Table 2, need frustration is important in the association with controlled forms of motivation that have received less attention in the literature, maybe because it has been challenging to predict by need satisfaction. Need frustration is, therefore, important to take into consideration when assessing the dark side of motivational processes at work.

### Limitations and Future Research Directions

Some potential limitations may exist in the present study. First, because the data were collected relying only on the BPNSFWS self-report measure to assess the internal process of need satisfaction and frustration, the results may have been influenced by common method variance (Podsakoff et al., 2003). Future studies might examine whether other methodological artifacts or personality factors may influence responses to the scale. Second, the present study supports the criterion-related validity of the BPNSFWS by means of cross-sectional associations. Future studies may further examine temporal stability of the measurement scale and causal relations between work-related need satisfaction and need frustration and its antecedents and consequences by means of longitudinal or experimental studies. In addition, diary studies can be used to focus on intra-individual differences in need satisfaction and frustration and their correlates. Third, the present study included convenience samples of different organization- or occupation-specific samples. Future research in different sectors and countries may further add to the generalizability of the findings. Moreover, while the present study, which was adapted and validated in Norwegian and English represents the first step toward a valid measurement instrument for use in the study of the work-related question in organizational psychology within the framework of SDT, validation of the scale in other languages will be fruitful. Lastly, in recent years the concept of need unfulfillment has emerged in the literature accounting for a possible third dimension of need states of relevance in considering the role of basic psychological needs in human motivation, functioning, and wellness (Huyghebaert-Zouaghi et al., 2020). Adding this dimension to the BPNSFWS to establish a common measurement of also this possible dimension of the basic psychological need can be worthwhile in future research efforts.

### Conclusion

SDT offers a framework for understanding motivational processes at work where fulfillment or frustration of employees' basic psychological needs are highlighted as key facilitators of employees' motivation, work functioning, and well-being. The current study provides researchers with a tool to assess employees' basic psychological need satisfaction and frustration at work by adapting the current go-to measurement instrument for basic psychological need satisfaction and need frustration to the work context. In addition, it validates the BPNSFWS in Norwegian and English. Relying on a common operationalization of need satisfaction and frustration in future research enables comparisons across studies, cultures, and contexts that contribute to a more unified development of this field. Therefore, the current study contributes to a unifying measurement of one of the central underpinnings of SDT.

## Data Availability Statement

The raw data supporting the conclusions of this article will be made available by the authors upon reasonable request.

## Ethics Statement

The studies involving human participants were reviewed and approved by Norwegian Center for Research Data. The patients/participants provided their written informed consent to participate in this study.

## Author Contributions

AO: responsible for collection of data, data analysis, and writing up the manuscript. HH: contributed in data collection and to writing up the manuscript. CWF: contributed in data collection and to writing up the manuscript.

## Conflict of Interest

The authors declare that the research was conducted in the absence of any commercial or financial relationships that could be construed as a potential conflict of interest.

## Footnotes

^1^Modification indices suggested adding residual covariances between some of the indicators for the six factors. In sample 1, 6 residual covariances were added (competence satisfaction item 1 with competence satisfaction item 2, relatedness satisfaction item 1 with relatedness satisfaction item 3, autonomy satisfaction item 1 with autonomy satisfaction item 3, autonomy frustration item 2 with autonomy frustration item 3, competence frustration item 2 with competence frustration item 4, relatedness frustration item 1 with relatedness frustration item 2). These residual covariances were included in the test of MI and represented group-specific parameters, meaning that partial MI is tested.

^2^Modification indices suggested adding residual covariances between some of the indicators for the six factors. In sample 2, 4 residual covariances were added (competence satisfaction item 1 with competence satisfaction item 4, autonomy frustration item 1 with autonomy frustration item 4, relatedness frustration item 1 with relatedness frustration item 2, autonomy satisfaction item 2 with autonomy satisfaction item 3). These residual covariances were included in the test of MI and represented group-specific parameters, meaning that partial MI is tested.

^3^Modification indices suggested adding residual covariances between some of the indicators for the six factors. In sample 3, 4 residual covariances were added (autonomy frustration item 2 with autonomy frustration item 3, relatedness satisfaction item 1 with relatedness satisfaction item 3, relatedness frustration item 1 with relatedness frustration item 2, relatedness satisfaction item 2 with relatedness satisfaction item 3). These residual covariances were included in the test of MI and represented group-specific parameters, meaning that partial MI is tested.

^4^Modification indices suggested adding residual covariances between some of the indicators for the six factors. In sample 4, 5 residual covariances were added (autonomy frustration item 1 with autonomy frustration item 4, relatedness satisfaction item 1 with relatedness satisfaction item 3, relatedness frustration item 1 with relatedness frustration item 4, competence frustration item 1 with competence frustration item 4, competence frustration item 3 with competence frustration item 4). These residual covariances were included in the test of MI and represented group-specific parameters, meaning that partial MI is tested.

^5^Modification indices suggested adding residual covariances between some of the indicators for the six factors. In the total Norwegian sample, 7 residual covariances were added (autonomy frustration item 2 with autonomy frustration item 3, relatedness satisfaction item 1 with relatedness satisfaction item 2, relatedness frustration item 1 with relatedness frustration item 3, competence satisfaction item 3 with competence satisfaction item 3, competence frustration item 1 with competence frustration item 4, relatedness satisfaction item 2 with relatedness satisfaction item 3, relatedness satisfaction item 1 with relatedness satisfaction item 2). These residual covariances were included in the test of MI and represented group-specific parameters, meaning that partial MI is tested.
