# Supplementary material for: The Basic Psychological Need Satisfaction and Need Frustration at Work Scale: A Validation Study
Source: Front Psychol. 2021 Jul 14;12:697306. doi: 10.3389/fpsyg.2021.697306 (PMC8316828; doi:10.3389/fpsyg.2021.697306)
Supplement: Supplementary file 1 [file Data_Sheet_1.pdf]

Appendix A

Skewness and kurtosis for the measurement items

	**Sample 1**		**Sample 2**		**Sample 3**		**Sample 4**	
	Skewness	*Kurtosis*	*Skewness*	*Kurtosis*	*Skewness*	*Kurtosis*	*Skewness*	*Kurtosis*
NA1	–0.873	0.103	–1.279	1.331	–0.884	0.369	-1.061	0.245
NA2	–0.754	0.309	–1.176	1.698	–1.060	1.432	-1.002	0.116
NA3	–0.628	0.155	–1.060	0.994	–0.770	0.437	-0.847	-0.285
NA4	–0.641	0.081	–1.113	1.667	–1.147	1.797	-0.796	-0.400
NC1	–1.240	3.300	–1.584	5.338	–1.924	6.831	-1.524	3.125
NC2	–1.598	5.819	–2.009	7.512	–1.578	6.423	-1.840	4.715
NC3	–1.212	3.773	–1.216	2.188	–1.025	2.114	-1.570	3.643
NC4	–1.225	3.478	–1.231	4.445	–1.263	3.101	-1.706	5.111
NR1	–0.830	0.581	–1.713	4.647	–1.238	1.975	-1.050	0.765
NR2	–0.659	0.055	–1.347	2.851	–0.916	0.736	-1.041	0.474
NR3	–0.580	0.171	–0.879	0.951	–0.639	0.249	-0.949	0.313
NR4	–0.792	0.640	–1.198	1.331	–1.080	1.374	-1.135	0.862
TNA1	0.022	–0.964	0.268	–1.054	0.011	–1.038	-0.389	-1.145
TNA2	0.567	–0.595	1.049	0.353	0.386	–0.818	0.184	-1.240
TNA3	0.834	–0.116	1.321	0.983	0.698	–0.350	0.135	-1.233
TNA4	0.600	–0.605	0.981	0.012	0.575	–0.621	-0.266	-1.194
TNC1	1.942	4.600	1.893	5.557	1.575	2.793	1.416	1.302
TNC2	1.112	0.608	2.026	5.424	1.481	2.183	1.294	0.904
TNC3	1.282	1.637	1.788	3.455	1.660	2.864	1.247	0.468
TNC4	1.680	2.643	1.904	3.895	1.897	3.963	1.334	0.831
TNR1	1.759	2.714	2.067	4.121	1.544	1.796	1.094	0.093
TNR2	2.020	4.452	1.992	3.713	1.496	1.512	1.167	0.307
TNR3	2.426	7.001	2.235	4.635	1.615	1.940	1.240	0.431
TNR4	1.233	0.945	1.158	0.444	1.120	0.429	0.472	-1.094

Appendix B

Item Means (M), Standard errors (SE), Factor Loadings (FL), and FL/SE

	**Sample 1**						**Sample 2**						**Sample 3**						**Sample 4**					
	M	SE	FL	FL/SE	α	ω	M	SE	FL	FL/SE	α	ω	M	SE	FL	FL/SE	α	ω	M	SE	FL	FL/SE	α	ω
Autonomy satisfaction					.88	.88					.84	.84					.81	.81					.91	.91
I have a feeling of choice and freedom in what I do at work	5.15	.045	.67	15.034			5.53	.036	.81	22.224			5.27	.032	.72	22.744			5.07	.028	.77	27.610		
I feel that the decisions I make at work reflect what I really want	5.02	.028	.85	30.119			5.42	.048	.79	16.567			5.31	.020	.86	42.399			5.11	.011	.92	85.037		
At work, I feel that the choices I make express who I really am	4.83	.018	.90	49.673			5.32	.056	.71	12.836			5.29	.026	.78	30.053			4.97	.019	.90	46.236		
At work, I feel that I do what really interests me	4.87	.028	.75	26.521			5.58	.04	.60	14.639			5.57	.036	.58	16.115			4.92	.020	.80	39.586		
Competence satisfaction					.88	.88					.84	.84					.83	.83					.87	.88
I feel confident that I can do things well at work	6.00	.051	.73	14.339			6.25	.054	.82	15.359			6.08	.034	.77	22.542			6.00	.030	.84	27.810		
I feel capable of doing what I do at work	6.08	.062	.83	11.635			6.30	.026	.77	29.136			6.19	.044	.70	15.718			6.12	0.21	.86	40.598		
I feel competent in reaching my goals at work	6.05	.025	.86	34.872			6.27	.053	.70	13.191			6.06	.030	.80	26.995			5.99	.030	.77	25.443		
I feel that I can successfully complete difficult tasks at work	5.88	.035	.84	23.971			6.05	.039	.82	21.035			5.88	.041	.68	16.566			6.05	.037	.72	19.613		
Relatedness satisfaction					.83	.83					.86	.87					.84	.85					.94	.94
I feel that the people I care about at work also care about me	5.86	.032	.77	23.726			6.14	.034	.80	23.765			5.91	.025	.80	31.523			5.36	.012	.91	73.953		
I feel connected to the people at work who care about me and whom I care about	5.69	.026	.83	31.898			5.95	.027	.86	32.227			5.72	.036	.72	20.177			5.27	.010	.92	88.636		
I feel closely connected to other people who are important to me at work	4.95	.038	.77	20.391			5.44	.056	.67	11.949			5.23	.049	.70	14.339			5.24	.016	.91	56.822		
I experience a warm and good feeling with the people I spend time with at work	5.35	.042	.72	17.255			5.67	.021	.86	41.333			5.47	.030	.78	26.073			5.28	.016	.86	52.927		
Autonomy frustration					.87	.87					.84	.83					.82	.82					.90	.90
Most of the things I do at work, I do because I feel that I have to	3.91	.043	.63	14.772			3.47	.043	.51	11.965			3.93	.037	.63	17.151			4.57	.030	.75	25.179		

At work, I feel forced to do many things that I would not have chosen to do	2.93	.032	.77	23.813		2.55	.021	.90	42.886		3.16	.038	.66	17.501		3.76	.018	.89	49.663	
I feel pressured to do many of the things I do at work	2.52	.030	.80	26.839		2.28	.019	.94	48.181		2.67	.035	.70	20.114		3.72	.016	.87	54.370	
My daily activities at work feel like a continuous line of duties	2.83	.021	.87	40.930		2.55	.043	.61	14.220		2.98	.032	.80	24.921		4.36	.030	.74	24.372	
Competence frustration					.78 79					.89 .90					.82 .82					.93 .90
I seriously doubt whether I can do things well at work	1.91	.062	.62	9.957		1.64	.030	.80	26.981		2.04	.026	.75	28.524		2.24	.012	.83	75.377	
I feel disappointment at many of my achievements at work	2.28	.043	.60	13.858		1.76	.027	.85	31.007		2.11	.036	.72	19.948		2.38	.022	.87	39.329	
I feel insecure about my abilities at work	1.94	.033	.81	24.238		1.80	.017	.89	51.139		1.98	.031	.75	23.951		2.41	.019	.86	44.930	
At work, I feel like a failure because of the mistakes I make	1.85	.041	.69	16.765		1.79	.038	.76	20.103		1.88	.033	.73	22.183		2.27	.020	.85	43.468	
Relatedness frustration					.86 .87					.90 .90					.88 88					.90 .90
At work, I feel excluded from the group that I want to be a part of	1.84	.045	.74	16.519		1.78	.040	.78	19.322		2.05	.037	.72	19.487		2.47	.025	.84	33.744	
At work, I feel that the people that are important to me are cold and distant towards me	1.72	.031	.82	26.787		1.73	.033	.84	25.674		1.95	.025	.85	33.870		2.47	.013	.91	72.222	
I have the impression that people that I spend time with at work dislike me	1.54	.048	.70	14.724		1.67	.030	.84	27.721		1.81	.025	.83	33.657		2.45	.016	.87	54.406	
I feel that the relations I have at work are only superficial	2.30	.030	.80	26.168		2.32	.025	.84	33.151		2.42	.022	.81	36.100		3.25	.021	.76	37.074	
